# Using Patient Experiences on Dutch Social Media to Supervise Health Care Services: Exploratory Study

**DOI:** 10.2196/jmir.3906

**Published:** 2015-01-15

**Authors:** Tom H van de Belt, Lucien JLPG Engelen, Lise M Verhoef, Marian JA van der Weide, Lisette Schoonhoven, Rudolf B Kool

**Affiliations:** ^1^Radboud REshape Innovation CenterRadboud University Medical CenterNijmegenNetherlands; ^2^Emergency Healthcare NetworkRadboud University Medical CenterNijmegenNetherlands; ^3^Scientific Institute for Quality of HealthcareRadboud Institute for Health SciencesRadboud University Medical CenterNijmegenNetherlands; ^4^The Healthcare InspectorateUtrechtNetherlands; ^5^Faculty of Health SciencesUniversity of SouthamptonSouthamptonUnited Kingdom

**Keywords:** social media, rating sites, patient safety, supervision

## Abstract

**Background:**

Social media has become mainstream and a growing number of people use it to share health care-related experiences, for example on health care rating sites. These users’ experiences and ratings on social media seem to be associated with quality of care. Therefore, information shared by citizens on social media could be of additional value for supervising the quality and safety of health care services by regulatory bodies, thereby stimulating participation by consumers.

**Objective:**

The objective of the study was to identify the added value of social media for two types of supervision by the Dutch Healthcare Inspectorate (DHI), which is the regulatory body charged with supervising the quality and safety of health care services in the Netherlands. These were (1) supervision in response to incidents reported by individuals, and (2) risk-based supervision.

**Methods:**

We performed an exploratory study in cooperation with the DHI and searched different social media sources such as Twitter, Facebook, and healthcare rating sites to find additional information for these incidents and topics, from five different sectors. Supervision experts determined the added value for each individual result found, making use of pre-developed scales.

**Results:**

Searches in social media resulted in relevant information for six of 40 incidents studied and provided relevant additional information in 72 of 116 cases in risk-based supervision of long-term elderly care.

**Conclusions:**

The results showed that social media could be used to include the patient’s perspective in supervision. However, it appeared that the rating site ZorgkaartNederland was the only source that provided information that was of additional value for the DHI, while other sources such as forums and social networks like Twitter and Facebook did not result in additional information. This information could be of importance for health care inspectorates, particularly for its enforcement by risk-based supervision in care of the elderly. Further research is needed to determine the added value for other health care sectors.

## Introduction

Social media has become a mainstream online tool that enables any individual to connect, create, consume, and control content, independently of time and place [1]. As a result, the Internet contains an abundance of user-generated content [2]. A growing number of people use social media for health care-related purposes such as finding health-related information. Research from the Netherlands showed that one in four adults would like to use social media to communicate with health care professionals [3]. Other studies demonstrated that an increasing number of patients share their experiences with health care providers or health care institutions via social media [4]. Experiences can also be shared by family [5]. This can be done using general social networks such as Facebook [6] or Twitter [7]. Other people use so-called “health care rating sites”, such as physician rating sites [8]. Health care rating sites allow users to rate and discuss individual health care providers or organizations. This can often be done very easily, anonymously, and free of charge. Not surprisingly, the number of sites and ratings is growing [9-11], and this may be boosted by the appearance of health care rating apps for mobile devices [12].

Not surprisingly, the scientific community has become increasingly interested in social media and health care rating sites, and many studies have been conducted on this topic. It is clear that health care rating sites may affect choices that people make about their health care [13], and could affect the way that patients and professionals interact. For example, negative reviews may result in new policies or could trigger doctors to change the way they communicate [14]. Information about health care on social media could also be useful to monitor the quality and safety of health care. An extensive review published in this journal confirmed that there is a relation between information on social media and traditional measures of quality of care. Verhoef et al analyzed 29 studies and showed that information derived from social media and health care rating sites correlated to several measures of quality of care including patient experiences, mortality, readmission rates, and infection rates [15]. More recently, research showed that messages on Twitter (tweets) may also contain unique information about patients’ experiences that cannot be acquired via other ways [16].

Although correlations are not always strong and several questions remain unanswered, social media seems to be a potential source of information about individuals’ perspectives of the quality and safety of care. If so, it could be useful for organizations such as health care inspectorates that want to make use of these individuals’ perspectives to supervise the quality and safety of care.

An example of such an organization is the Dutch Healthcare Inspectorate (DHI), an agency under the Ministry of Health, Welfare and Sport. It is the official regulatory body charged with supervising the quality and safety of health care services, prevention activities, and medical products in the Netherlands. The DHI has organized its supervision in several ways to ensure compliance with (professional) standards and guidelines and to ensure patient safety. The two most important ways are (1) incident-based supervision, and (2) analyses of various types of risk information, also known as risk-based supervision [17]. For several reasons, supervisory bodies such as the DHI might be interested in patient experiences shared on social media. First, health care inspectorates have the responsibility to supervise a huge number of health care suppliers, which makes it difficult to collect information. For example, the DHI supervises around 40,000 health care-related institutions and 800,000 health care professionals [18]. Therefore, social media could be an additional source of information that enables the DHI to prioritize and supervise organizations in a more efficient way. Second, the DHI is looking for more current information, since there is usually a time lag between the actual incident itself and the availability of information about the incident for health care inspectors from conventional sources such as care-related quality indicators. For example, quality indicators are usually collected only once a year. A third goal to pursue regarding information on social media is to stimulate citizen or consumer participation in supervising the quality and safety of health care services [19]. This has gained popularity in the past few years and can result in valuable information [20,21]. Therefore, in close cooperation with relevant health care inspectorate employees of the DHI, we explored the following research question: What is the added value of information on social media for supervising health care services, in both incident-based and risk-based supervision?

## Methods

### Design and Setting

To the best of our knowledge, the use of social media in supervising the quality and safety of health care services by health care inspectorates has not been studied intensively. Consequently, we performed an exploratory study, which consisted of two parts. The first part was aimed at identifying added value of social media, for supervision based on incidents reported to the health care inspectorate. We searched social media sources to find additional information about incidents for which insufficient information was available. Health care inspectors determined the potential value of the information found. The second part of this study was focused on the added value of social media for risk-based supervision after assessment of specific risks of health care providers. We searched social media to find additional information to identify whether it could function as an indicator for high-risk providers. Experts from the health care inspectorate determined the potential additional value of the information found.

### Selection of Social Media Sources and/or Monitoring Tools

First, we identified usable, efficient, and reliable social media sources or tools to search social media. Social media sources consisted of individual original sources such as Twitter or Facebook, and tools consisted of standard search engines such as Google or specific social media monitoring tools (SMMTs). SMMTs are tools that allow users to search several social media sources at the same time. Social media sources and tools to search social media were selected by going through three operational steps. First, two researchers with past experience in performing online searches in academic and grey literature, determined the following inclusion criteria for use in this study: (1) related to the Dutch language, (2) ability to search for messages posted up to at least 2 years back, since the DHI takes into account reports about incidents that took place within the past 2 years, (3) accessible, since private social networks are inaccessible, and (4) supporting Boolean search. The latter is a technique that allows the creation of specific search strings making use of operators like “AND”, “NOT”, and “OR” [22].

Second, we searched for search tools on the Internet and asked experts to share their best options. We also screened an existing online list of SMMTs for relevant tools [23]. Furthermore, we used information and experiences about SMMTs obtained during previous projects performed by the DHI. We created a list of potentially relevant tools and verified that they met pre-defined inclusion criteria.

Third and last, test searches were performed with all social media sources and/or tools that corresponded to the criteria. Two researchers individually performed three test searches for randomly selected health care providers. They developed a search strategy to find any information about this health care provider that was available online, such as experiences or ratings. Relevant features and/or findings were added to a table and results and experiences were discussed together. This step not only allowed the researchers to get acquainted with the selected tools, but also to identify any issues regarding reliability, specificity, or usability. For example, if a tool was hosted on a website that was extremely slow and if it was clear that this problem could not be solved, the researchers could decide to exclude the tool. For practical reasons, we aimed at selecting three to five of the most suitable social media sources and/or tools for use in this study. For the second search, we selected social media sources and/or tools based on the results of the first search and removed sources or tools that had not resulted in any new information from the list.

### Searches for Information for Incident-Based Supervision

We started by creating searches for 20 reported incidents, which were randomly selected from the DHI database. Random selection was needed to keep a wide focus and to assure that the analysis could apply to different sectors such as elderly care, hospital care, and non-hospital care. Two researchers (TB and LV) individually studied the summaries of the first five reported incidents and created search queries for each incident. Summaries were found suitable for this purpose since they contained all information needed for this study, including a description of the incident, date, name of the person that reported the incident, health care provider, specialty, and organization. For each search strategy, the goal was to find additional information (within social media sources) related to the reporting individual, the disease or treatment, the health care provider, and/or the organization that could be of value for the DHI in determining the relevance of the reported incident. We then tailored the search queries to each social media source or tool, since every source or tool had different options such as rules and use of search operators. To reduce inter-researcher variability, the researchers discussed and improved the search queries and results for the first five searches, until they both agreed on the search query.

Next, one of the two researchers (TB) created search queries for the 15 incidents remaining and performed the searches. Any issues were resolved by consulting another researcher (LV). Furthermore, the second researcher screened search queries, in order to make sure that the basics of each query were identical and operators (eg, AND, NOT) were used in the right way. Print-screens were made for all searches. In the case of many hits, only print-screens of the first two pages with results were saved.

Finally, all results related to the original reported incident were summarized in a text file and shared with DHI inspectors. Per sector, one inspector determined the additional value (eg, information leads to specific actions or may influence a decision) of the information found. They were asked to choose between “0: No additional value of the information found” or “ 1: Information is relevant and contains additional information”. Furthermore, an explanation of each answer was retrieved.

We also performed searches for incidents after purposive sampling: four reported incidents in one of the five major health care sectors to retain a wide focus (hospital care, primary care, mental care, long-term care, and home care). We asked the DHI to select incidents for which it was unclear whether action by the health care inspectorate was necessary. The search queries were developed and results were summarized using the same procedure as the first search. Since the first round taught us that two options for categorization did not provide enough differentiation, we added two categories. Inspectors could determine the relevance of all information found by selecting one of the following options: Relevant, information leads to immediate action (3), Relevant, information leads to further investigations (2), Relevant, information found leads to a signal in the file of the organization (1), or No additional value (0). A description of each option is provided in Multimedia Appendix 1.

### Searches for Information for Risk-Based Supervision

First, we asked DHI experts to select high-risk themes for which additional information was preferred. Second, one researcher (TB) developed a search query for each high-risk theme, which was peer-reviewed by a second researcher (LV). After discussion and improvement, a DHI expert working on the selected high-risk themes peer-reviewed the queries, which resulted in the final search strategies. Third, we performed searches for the high-risk themes and presented results in a spreadsheet. In case a result concerned a rating, the ones without a textual description or ratings ≥7 (on a scale from 1 “extremely bad” to 10 “excellent”) were excluded. The latter was done since we reasoned that the useful information for inspectorates would be found in ratings <7. Subsequently, both researchers independently filtered results based on relevance by reading individual results and comparing it to the search query. All results for which no relation to the search could be determined were excluded. For example, if the search included “long-term elderly care”, results about providers working in other sectors such as care delivery by a local GP were excluded. Fourth and last, one expert per theme determined the relevance of each result following the same procedure and the same options as described above and in Multimedia Appendix 1.

### Ethical Approval

Since this study used anonymous data from the public domain and without patient involvement, no ethical approval was needed in the Netherlands. More specifically, all data we used from the rating site ZorgkaartNederland.nl are publicly available. Furthermore, we obtained permission from the DHI to perform the study and acquired the data needed for this study.

## Results

### Incident-Based Supervision

We identified 11 possible tools that could be used to find additional information for incident-based supervision. Based on each tool’s features, we selected four tools to perform our searches: Coosto, Google, Addictomatic, and ZorgkaartNederland. Further information about each tool’s features and the selection of tools is provided in Multimedia Appendix 2.

Table 1 shows the results of the searches for incident-based supervision. The 20 searches performed for randomly selected incidents (Round 1) resulted in additional information for six cases. Following review by the inspectors of the DHI, the additional information led to adding a “signal” in three cases. A signal implies that information about specific cases or issues is added to the DHI’s files, allowing the DHI to keep track of relevant issues over time. All relevant results were found via the source ZorgkaartNederland, a major Dutch rating site. Therefore, we only consulted ZorgkaartNederland in Round 2. The 20 searches performed for the non-randomly selected incidents resulted in additional information for three cases. After assessment, the additional information found led to adding a signal in two cases. In one case, the information found led to further investigation into the case.

**Table 1:** Added value of information for incident-based supervision.

	Incident-based supervision, Round 1 (n=20)	Incident-based supervision, Round 2 (n=20)
Immediate action required	0	0
Information leads to further research	0	1
Information leads to signal	3	2
Information found but no added value	3	0
No information found	14	17

### Risk-Based Supervision

Regarding the searches for risk-based supervision, the DHI selected the high-risk sector long-term elderly care, combined with four specific themes that form major safety risks: hygiene, professional expertise, medication safety, and restriction of freedom. Based on results from the searches for incident-based supervision, Coosto was selected as the preferred tool to perform all four searches. ZorgkaartNederland was selected as the preferred source since the first part taught us that it was the only source that provided us with relevant results. Table 2 provides detailed information about the number of hits, ratings that remained after exclusion, and the results after assessment by a health care inspector. The added value of the information varied between the four themes: restriction of freedom (100%, 2/2), hygiene (88%, 22/25), medication safety (76%, 16/21), and expertise (47%, 32/68).

**Table 2:** Added value of information for risk-based supervision.

		Indicator 1: Long-term elderly care and hygiene	Indicator 2: Long-term elderly care and expertise	Indicator 3: Long-term elderly care and medication (safety)	Indicator 4: Long-term elderly care and restriction of freedom
		n (%)			
Hits (n)		79 (100%)	117 (100%)	49 (100%)	11 (100%)
Results remaining after exclusion of ratings 7 or higher		38/79 (48%)	90/117 (77%)	34/49 (69%)	7/11 (64%)
Results remaining for assessment by health care inspector after exclusion ratings not related to search query		25/79 (32%)	68/117 (58%)	21/49 (43%)	2/11 (18%)
**Results after assessment by DHI**					
	Immediate action required	0/25 (0%)	0/68 (0%)	0/21 (0%)	0/2 (0%)
	Information leads to further research	9/25 (36%)	1/68 (1%)	1/21 (5%)	0/2 (0%)
	Information leads to signal	13/25 (52%)	31/68 (46%)	15/21 (71%)	2/2 (100%)
	No added value of the information found	3/25 (12%)	36/68 (53%)	5/21 (24%)	0/2 (0%)

## Discussion

### Principal Findings

In this study, we showed that a Dutch health care rating site can be used to identify additional information for supervising quality and safety, especially in long-term elderly care. These findings indicate that social media may enable supervisory bodies to include the patients’ perspective in a more efficient way. Regarding incident-based supervision, social media provided relevant additional information in six of 40 incidents, and for risk-based supervision, social media provided relevant additional information in 72 of 116 cases. Additional information led to a signal or to further research. Although these numbers are promising for supervisory bodies looking for an efficient way of collecting information from the patients’ perspective on health care providers, several things need to be discussed.

In keeping with our approach, we aimed at including all Dutch social media sources. It appeared that the rating site ZorgkaartNederland was the only source that provided information that was of additional value for the DHI. It may seem surprising that only one social media source, among the plethora of other sources such as forums and social networks like Twitter and Facebook, resulted in additional information. Apparently, Dutch people are not likely to share their experiences with health care in combination with the name of their health care provider in tweets or public Facebook posts. Regarding ZorgkaartNederland, it should be considered that it is the only Dutch website with a list of all officially registered health care providers and organizations, which aims to collect as many ratings as possible. Furthermore, ZorgkaartNederland is a non-commercial initiative supported by the Dutch Federation for Patients and Consumers. As a result, people willing to share a rating or their experiences are likely to use ZorgkaartNederland. There are hardly any serious competitors. Therefore, ZorgkaartNederland could be, at least at the present time, a valuable source of information for the DHI. It would be worthwhile to explore the possibility of creating a direct link between relevant social media sources such as ZorgkaartNederland and the DHI (eg, via an open API), allowing the DHI to make a selection of ratings available for use in daily practice. In fact, the DHI is already implementing a system in which information from ZorgkaartNederland is imported into a risk database for its daily supervision.

In this study, we performed searches for the two most used instruments by health care inspectorates: supervision in response to reported incidents and risk analysis. The results show that the searches for risk-based supervision resulted in more relevant information than searches for reported incidents. This can be explained by the search goals for every search. Regarding searches for supervision based on reported incidents, searches were created using the information from the reported incident only, and we aimed to find information that could indicate similar events or structural problems regarding one provider or organization only. Regarding the searches for supervision led by risk analysis, we created searches aimed at finding relevant information about a specific theme in a group of health care providers or organizations within an entire sector. The added value of the information varied strongly: restriction of freedom (100%), hygiene (88%), medication safety (76%), and expertise (47%). This might be explained by the extent in which lay people are able to judge health care situations (eg, it is easy to see that toilets are dirty, but it is harder to determine whether you were given the correct dosage of medication). Searching for additional information for an entire theme with one search (risk analysis) is less time consuming than creating a unique search for every individual case (reported incidents).

### Future Research

In the present study, we performed searches for risks in four sectors in part one and one sector in part two. Therefore, future projects should investigate the generalizability to other health care sectors. This seems to be particularly relevant since the use of rating sites may be different for different demographical groups [24]. In contrast to the present study in which we used social media in a passive way, we feel that it would also be worthwhile to explore the use of social media in an active way, especially since social media facilitate interaction [1]. Besides, some other Dutch inspectorates already use social media interactively. For example, the Dutch Inspectorate for Social Affairs and Welfare uses a mobile phone app to enable consumers to share information about local asbestos conditions [25]. Furthermore, the Dutch Food and Consumer Product Safety Authority crowdsources information about plants that cause hay fever [26]. A hypothetical way of interactive use of social media by the DHI is actively requesting information from for example staff, patients, or relatives via social media, in preparation for inspections.

### Limitations

The use of social media for supervision of health care has some limitations, such as the lack of sufficient information, which has been recognized before [27]. A minimum amount of information is needed to provide robust predictions. Although this is investigated for tweets and not for ratings, it seems likely that the similar principle goes for ratings. As a result, there may be sectors with insufficient numbers of ratings to be of value for the DHI. Therefore, caution is advised and it is important to verify the number of ratings. A second limitation is related to our study design. Following our approach, only one expert per sector or theme determined the added value of social media. Since we have discussed the results with several other DHI experts, we think this has not affected our results significantly. However, future studies should aim to determine the value of the information based on at least two DHI experts by, for example, determining the inter-rater variability. A third limitation is related to the timeliness of the results found. In this study, only the source ZorgkaartNederland provided relevant information. In the Internet era, social media networks and rating sites can rapidly appear or disappear. Therefore, other sources can become more relevant for the DHI in the near future, and future studies should aim to keep their focus wide to assure that all relevant sources are searched. Another opportunity to look for information could be to explore specific (popular) sources with tailor-made research designs. This has recently been done for Twitter [16]. Last, we add that the generalizability of this study to other countries should also be explored since every country has its own systems of supervision and rating sites will be different.

### Conclusion

We conclude that social media could be used to include the patient’s perspective in supervision. This information could be of importance for health care inspectorates, particularly for its enforcement by risk-based supervision of elderly care. Further research is needed to determine the added value for other health care sectors.

## Multimedia Appendix 1

Scoring options to indicate the value of the information found.

## Multimedia Appendix 2

Features of the potential search tools.

## Abbreviations

DHI: Dutch Healthcare Inspectorate
SMMT: social media monitoring tool

## References

[1] Hoffman DL, Novak TP (2012). Why Do People Use Social Media? Empirical Findings and a New Theoretical Framework for Social Media Goal Pursuit. SSRN Journal.

[2] Van De Belt TH, Engelen LJ, Berben SA, Schoonhoven L (2010). Definition of Health 2.0 and Medicine 2.0: a systematic review. J Med Internet Res.

[3] Van de Belt TH, Engelen LJ, Berben SA, Teerenstra S, Samsom M, Schoonhoven L (2013). Internet and social media for health-related information and communication in health care: preferences of the Dutch general population. J Med Internet Res.

[4] Greaves F, Ramirez-Cano D, Millett C, Darzi A, Donaldson L (2013). Harnessing the cloud of patient experience: using social media to detect poor quality healthcare. BMJ Qual Saf.

[5] Adams S (2013). Post-panoptic surveillance through healthcare rating sites. Information, Communication & Society.

[6] Greene JA, Choudhry NK, Kilabuk E, Shrank WH (2011). Online social networking by patients with diabetes: a qualitative evaluation of communication with Facebook. J Gen Intern Med.

[7] Antheunis ML, Tates K, Nieboer TE (2013). Patients' and health professionals' use of social media in health care: motives, barriers and expectations. Patient Educ Couns.

[8] Lagu T, Hannon NS, Rothberg MB, Lindenauer PK (2010). Patients' evaluations of health care providers in the era of social networking: an analysis of physician-rating websites. J Gen Intern Med.

[9] Gao GG, McCullough JS, Agarwal R, Jha AK (2012). A changing landscape of physician quality reporting: analysis of patients' online ratings of their physicians over a 5-year period. J Med Internet Res.

[10] Greaves F, Millett C (2012). Consistently increasing numbers of online ratings of healthcare in England. J Med Internet Res.

[11] Hanauer DA, Zheng K, Singer DC, Gebremariam A, Davis MM (2014). Public awareness, perception, and use of online physician rating sites. JAMA.

[12] Bidmon S, Terlutter R, Röttl J (2014). What explains usage of mobile physician-rating apps? Results from a web-based questionnaire. J Med Internet Res.

[13] Greaves F, Pape UJ, King D, Darzi A, Majeed A, Wachter RM, Millett C (2012). Associations between Internet-based patient ratings and conventional surveys of patient experience in the English NHS: an observational study. BMJ Qual Saf.

[14] López A, Detz A, Ratanawongsa N, Sarkar U (2012). What patients say about their doctors online: a qualitative content analysis. J Gen Intern Med.

[15] Verhoef LM, Van de Belt TH, Engelen LJ, Schoonhoven L, Kool RB (2014). Social media and rating sites as tools to understanding quality of care: a scoping review. J Med Internet Res.

[16] Greaves F, Laverty AA, Cano DR, Moilanen K, Pulman S, Darzi A, Millett C (2014). Tweets about hospital quality: a mixed methods study. BMJ Qual Saf.

[17] IGZ website.

[18] IGZ organisatie.

[19] Robben P (2011). Burgerperspectief van de IGZ [Internal memo]. Utrecht: IGZ.

[20] Adams SA, van de Bovenkamp H, Robben P (2013). Including citizens in institutional reviews: expectations and experiences from the Dutch Healthcare Inspectorate. Health Expect.

[21] Rozenblum R, Bates DW (2013). Patient-centred healthcare, social media and the internet: the perfect storm?. BMJ Qual Saf.

[22] Wiki search engine.

[23] A Wiki of Social Media Monitoring Solutions.

[24] Terlutter R, Bidmon S, Röttl J (2014). Who uses physician-rating websites? Differences in sociodemographic variables, psychographic variables, and health status of users and nonusers of physician-rating websites. J Med Internet Res.

[25] Inspectie SZW.

[26] DFCPSA.

[27] Broniatowski DA, Paul MJ, Dredze M (2013). National and local influenza surveillance through Twitter: an analysis of the 2012-2013 influenza epidemic. PLoS One.

