# Supplementary material for: Using Patient Experiences on Dutch Social Media to Supervise Health Care Services: Exploratory Study
Source: J Med Internet Res. 2015 Jan 15;17(1):e7. doi: 10.2196/jmir.3906 (PMC4319082; doi:10.2196/jmir.3906)
Supplement: Supplementary file 1 [file jmir_v17i1e7_app1.pdf]

Additional file 1:

Score	Description
0 The rating has no added value for the DHI.	• Information is not relevant for the DHI<br>• Information is too concise to assess<br>• Information is not specific enough<br>• Information is contradictory or unclear
1 Based on this rating, a signal is added to the healthcare provider or organization.	• Information is relevant for the DHI<br>• Unclear whether it concerns an incident or a structural problem<br>• In case of similar signals, intervention is needed by the DHI.<br>• If this healthcare provider or organization will be visited in the near future, it is relevant to discuss this issue.
2 Based on this rating, the DHI will further investigate this issue.	• Information is relevant for the DHI<br>• Information may indicate a structural problem<br>• Safety of client/patient is in jeopardy and/or observed norm exceeding behaviour by a healthcare provider and/or media-sensitive issue<br>• Based on this information it is justified to ask for clarification (written) or to visit the organization
3 Based on this rating, the DHI will undertake immediate action.	• Information is relevant for the DHI<br>• Based on this information, immediate intervention is required.<br>• This situation comprehends direct risk of serious hazard or injury of client/patient and/or humiliating situations and/or media-sensitive issue.<br>• Program director is directly informed since this concerns a

serious situation.
