# Supplementary material for: Using Patient Experiences on Dutch Social Media to Supervise Health Care Services: Exploratory Study
Source: J Med Internet Res. 2015 Jan 15;17(1):e7. doi: 10.2196/jmir.3906 (PMC4319082; doi:10.2196/jmir.3906)
Supplement: Supplementary file 2 [file jmir_v17i1e7_app2.pdf]

Additional file 2:

	Focused on Dutch language?	Boolean search available?	Searches > 1 year?	Tested by DHI and not selected?	Already indexed by other sources	Judgement	Used in study
Coosto	Yes	Yes	Yes	No	No	++	Yes
Google	Yes	Yes	Yes	No	No	++	Yes
Addict-O-Matic	No	No	unknown	No	No	+/-	Yes
ZorgkaartNederland	Yes	No	Yes	No	Yes	+/-	Yes
Buzzcapture	Yes	Yes	No	No	No	-	No
Facebook	Yes	No	Yes	No	Yes	-	No
Twitter	Yes	No	Yes	No	Yes	-	No
Hootsuite	Yes	unknown	unknown	No	Yes	-	No
Radian 6	No	unknown	unknown	No	No	-	No
Clipit	Yes	unknown	unknown	Yes	No	-	No
Buzztalk	Yes	unknown	unknown	Yes	No	-	No
